# Correction of anterior bowing complicating tibial osteofibrous dysplasia in preadolescents by osteotomy and telescopic nailing without lesional resection: a preliminary study of four-case reports

**DOI:** 10.1186/s12891-024-07273-9

**Published:** 2024-02-19

**Authors:** Yanhui Jing, Zhiqiang Zhang, Yueqiang Mo, Dahui Wang, Chunxing Wu, Bo Ning

**Affiliations:** Department of Orthopaedics Surgery, National children’s medical center & Children’s Hospital of Fudan University, Wanyuan Road 399, Minhang District, Shanghai, 201102 China

**Keywords:** Tibia osteotomy,Telescopic Rod, Osteofibrous Dysplasia, Bowing Deformity

## Abstract

**Background:**

Osteofibrous dysplasia (OFD) occurs most frequently in the tibia and may result in deformity and pathological fracture. Surgical treatment such as curettage or segment excision has been performed but remains controversial due to high complication rates and surgical burden. This study introduces a new method to manage OFD with anterior bowing of the tibia using minimally invasive tibial osteotomy and telescopic rod (TR) osteosynthesis without extensive lesion resection.

**Methods:**

A retrospective study of 4 children with OFD and tibia bowing deformity treated with minimally invasive tibial wedge osteotomy and TR fixation between January 2015 and November 2020 was performed. Results including bone healing, complications, function based on MSTS score, and recurrance of deformity were assessed.

**Results:**

The median follow-up was 29 months. Radiographs showed the median time for union was 3 months. There were no instances of refracture or recurrence of deformity. The mean post-operative MSTS score was significantly higher than preoperative score.

**Conclusions:**

This method avoids large bone defects and reconstructive procedures. It is an effective and minimally invasive approach for managing anterior bowing deformity secondary to OFD while improving function and quality of life.

**Level of evidence:**

Level IV; Case Series; Treatment Study.

## Introduction

Osteofibrous dysplasia is a rare developmental, tumor-like condition distinct from fibrous dysplasia. Frangenheim first identified this condition in the tibia in 1921, terming it “congenital osteitis fibrosa” [1]. In 1976, Campanacci coined the term “osteofibrous dysplasia” to describe lesions bearing resemblances to fibrous dysplasia [2]. OFD constitutes 0.2% of primary bone tumors. The condition primarily affects the diaphysis of the tibia and, less frequently, the distal fibula, radius, and ulna [3].

Patients with OFD typically present with symptoms such as pain, pathological fractures, tibial swelling, or anterior bowing deformities. Recurrent fractures and tibial deformities can significantly impact the quality of life, especially in young children [4, 5]. While surgery remains the primary treatment for children with recurrent fractures or tibial deformities, focusing on lesion removal and preserving the unaffected bone, its application remains debated. This controversy stems from the potential for spontaneous lesion regression and the high recurrence rates post-surgery. Additionally, addressing extensive bone defects resulting from lesion resection poses a considerable challenge, often leading to significant complications, and imposing social and economic burdens even when various reconstructive procedures are implemented [6].

Recent research by Dala-Ali and Daniel Westacott suggests that OFD is a benign bone condition with low local progression rates and no malignant transformation risk. They argue that surgical interventions should prioritize addressing the deformity over the lesion [6, 7]. Our report introduces a novel approach to managing OFD using minimally invasive tibia osteotomy combined with telescopic rod osteosynthesis. This method aims to restore stability and alignment without lesion excision. We sought to evaluate the clinical outcomes of patients treated with this technique.

### Patients and Methods

We reviewed records from our hospital database of patients diagnosed with OFD between January 2015 and December 2020. We selected those with anterior bowing deformities who underwent minimally invasive osteotomy and TR fixation. Clinical attributes, including age, symptoms, lesion characteristics, postoperative outcomes, and follow-up data such as the MSTS score, were collated. This study received approval from our institutional ethics committee. Comprehensive clinical and follow-up data are presented in Tables 1 and 2.

**Table 1:** Patient Demographics and Clinical Data

Case	Sex	Age(years)	Site	Side	Segmental Length (cm)	Lesion Length Ratio (%)	Affected Part	Presenting Symptom	Angle Deformity
1	M	11	Tibia	Left	21.5	75	Circumferential	Anterior bowing, Persistent pain	48
2	M	11	Tibia	Left	16	50	Circumferential	Anterior bowing, Persistent pain	25
3	F	8	Tibia	Right	9	46	Circumferential	Anterior bowing, Persistent pain	25
4	M	8	Tibia&Fibia	Left	12	40	Circumferential	Anterior bowing Persistent pain	40

**Table 2:** Surgical Details and Follow-up Data

Case	Initial Treatment (Age at Initial Treatment)	Biopsy	Type of Operation	Union Time(Months)	Follow-up(Months)	Complication	Extension of TR(mm)	LLD (mm)	MSTS
1	Curettage+Allograft+ESIN aged (4 y)	OFD	Two segmental osteotomy	2.5	27	Wound infection	4	10	25
2	Curettage+Allograft+ESIN aged (5 y)	OFD	Two segmental osteotomy	3	31	Telescopic rod disaplacement.	2	7	28
3	Curettage+Allograft+EIIN aged (5 y)	OFD	Wedge osteotomy	2	32	none	16	8	27
4	Open obisy+brace aged (4 y)	OFD	Wedge osteotomy	4.5	24	none	20	5	26

### Surgical Method

All procedures were conducted under general anesthesia. The patient was positioned on a radiolucent operating table to facilitate intraoperative C-arm X-ray utilization. An anterolateral tibial incision was made across the tibial arch's apex (Fig. 1c). Subcutaneous tissue and fascia were sequentially incised to expose the periosteum. A wedge osteotomy was executed anteriorly using a power saw to rectify diaphyseal anterior deformities (Fig. 1d). The osteotomy was adjusted based on the specific case requirements, with the objective of correcting coronal, sagittal, and rotational deformities while minimizing bone exposure. Importantly, the osteotomy site lies within the tumor lesion, ensuring minimal excision if the osteotomy proves satisfactory. In our cohort, two tibial osteotomies were performed in cases 1 and 2 (Fig. 2), while cases 3 and 4 underwent a single osteotomy (Fig. 3). Pathological examinations were conducted during surgery to achieve clear pathological results. After achieving the desired tibial alignment, TRs were implanted, ensuring proper positioning using fluoroscopy. The surgical wound was closed in layers, and osteotomy samples were sent for pathological evaluation, which consistently showed no malignancy indications.

**Fig. 1:**
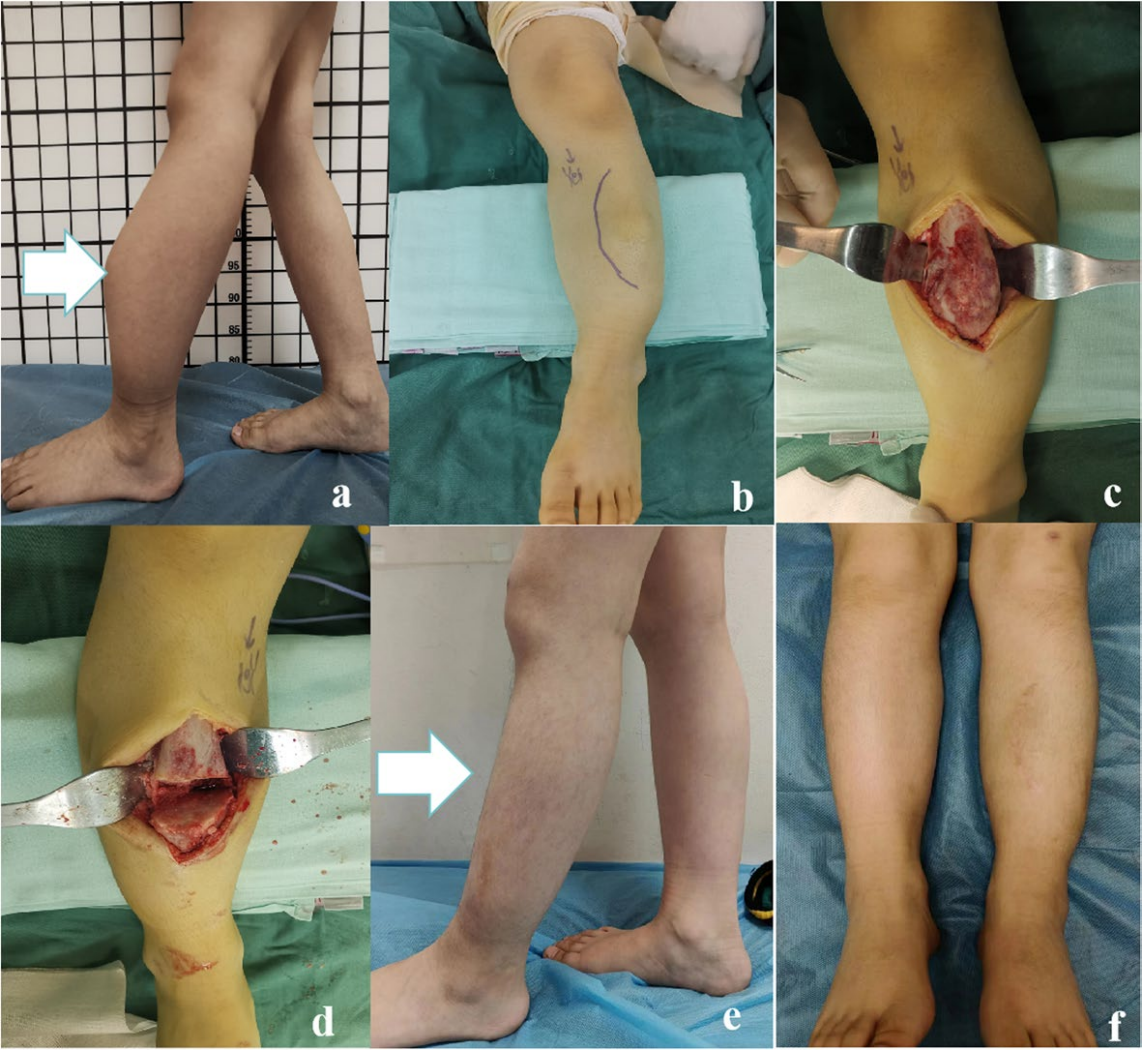
Surgical Procedure and Imaging for an 8-Year-Old Boy's Left Tibia. **a**,** b** Preoperative appearance of the left tibia, displaying a pronounced anterior bowing deformity. **c**,** d** Intraoperative photographs, showcasing the performance of an anteriorly based wedge osteotomy. **e**,** f** Postoperative appearance of the tibia, one year later, demonstrating successful correction of the bowing deformity

**Fig. 2:**
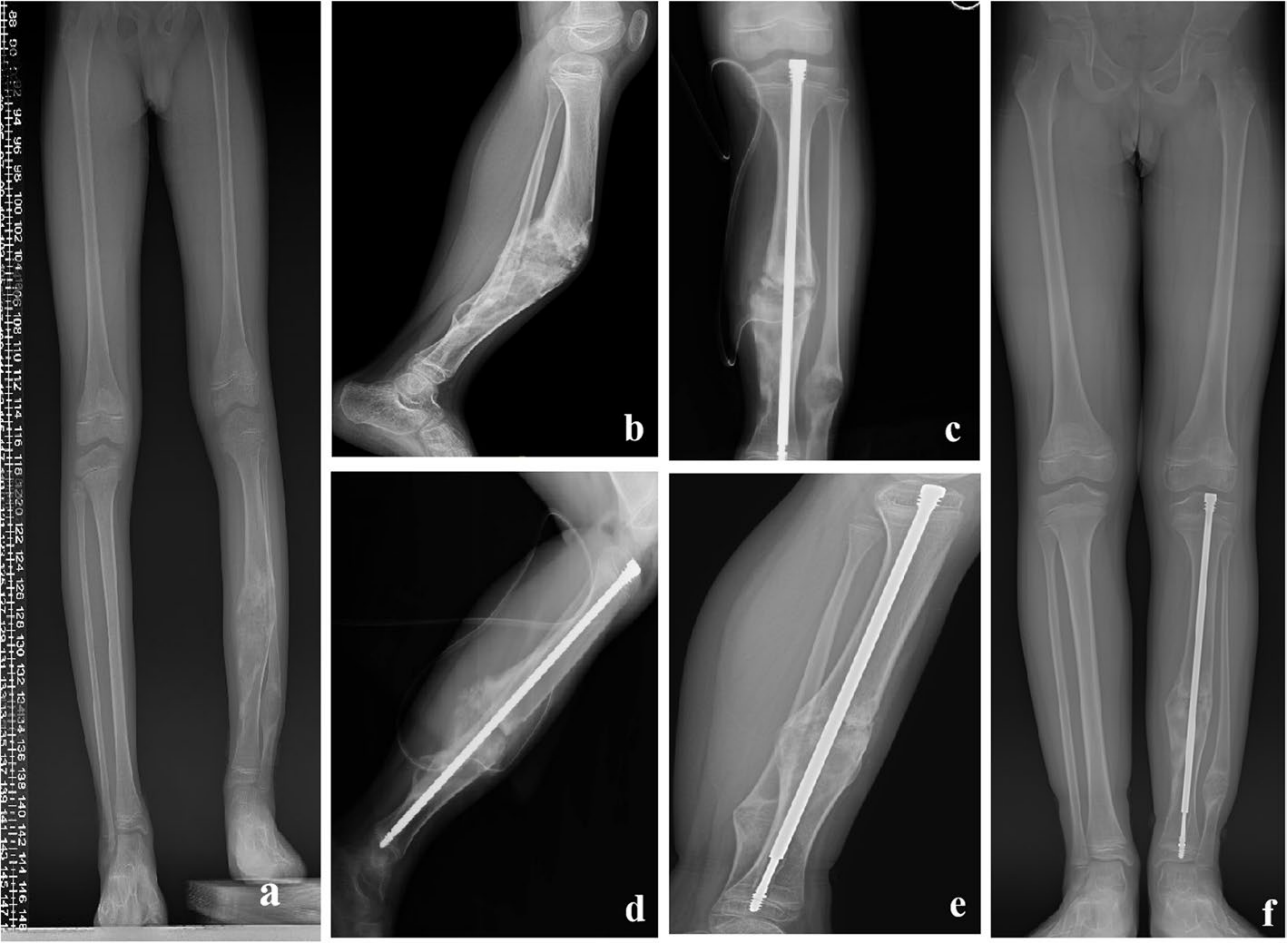
Outcomes for an 11-Year-Old Boy with OFD of the Left Tibia. **a**,** b** Anteroposterior (**a**) and lateral (**b**) radiographs obtained preoperatively, indicating a 48-degree anterior bowing deformity in the lateral view. **c**,** d** Intraoperative anteroposterior (**c**) and lateral (**d**) radiographs, displaying the wo tibial osteotomies performed. **e**,**f**, **g** Radiographs taken two years postoperatively, revealing sound bone healing, absence of deformity, and no implant failures. **c**,** d**, **e**, **f** the specific details of the distal tip of the nail in the postoperative and 2-year follow-up images,4-mm prolongation was present

**Fig. 3:**
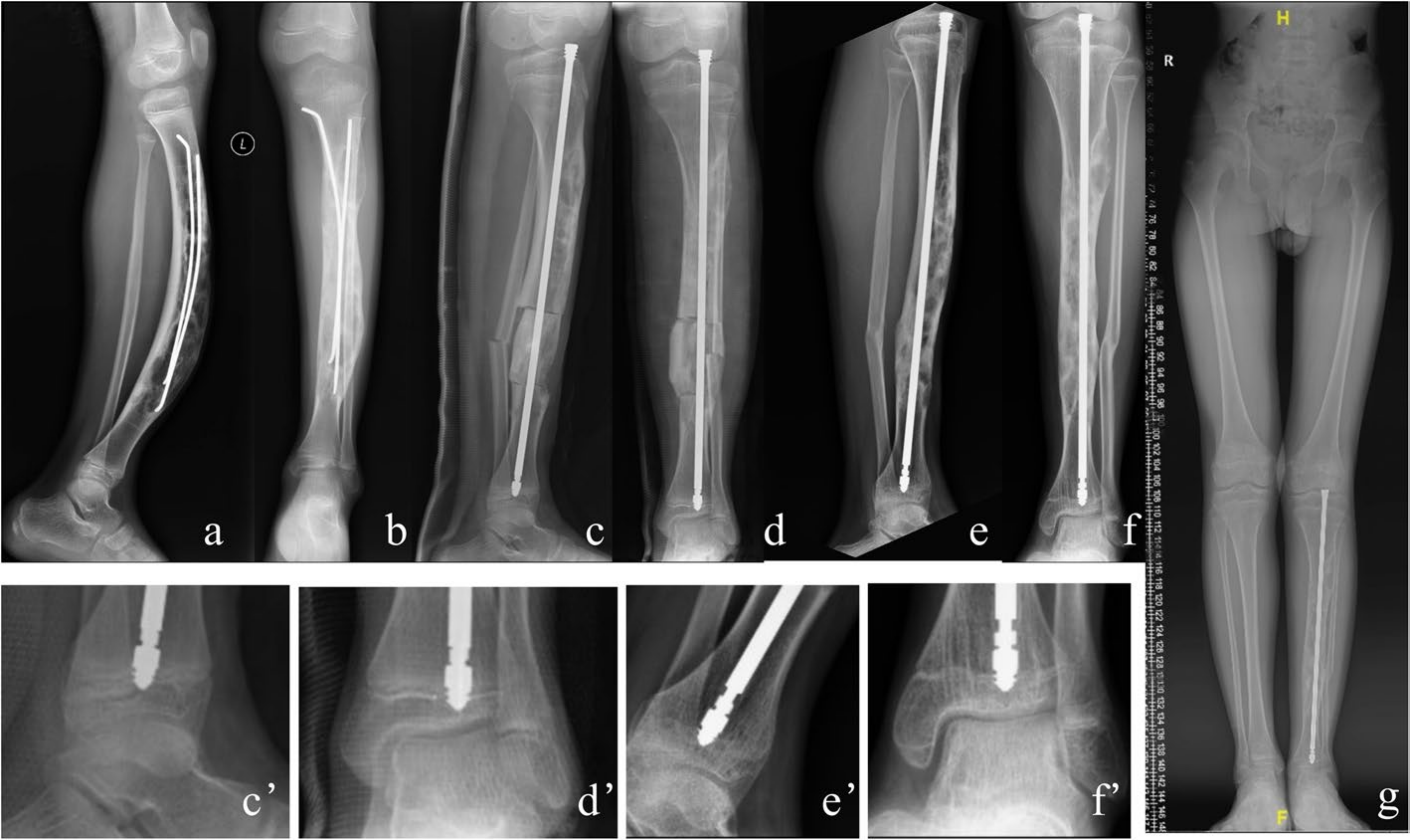
Results for an 8-Year-Old Boy with OFD of the Left Tibia. **a**,** b** Anteroposterior (**a**) and lateral (**b**) radiographs taken preoperatively reveal 40-degree anterior bowing deformity in the lateral view. **c**,** d** Intraoperative anteroposterior (**c**) and lateral (**d**) radiographs illustrating the single tibial osteotomy performed. **e**,** f** Lateral radiograph (**e**) and full-length limb X-ray (f) taken one year postoperatively, confirming normal limb alignment and absence of any significant discrepancy

If the fibula interferes with correcting the tibia, performing tibiofibular osteotomy concurrently is advisable.In our study, one case underwent tibiofibular osteotomy at the same time.

### Postoperative Management

Patients received prophylactic antibiotics for 48 hours post-surgery. Negative pressure drainage was removed on the second postoperative day. X-rays were taken bi-weekly, followed by long-leg casting once wound healing and swelling reduction were confirmed. Regular follow-ups were scheduled monthly post-discharge and quarterly post-fracture healing and cast removal. Complications, healing duration, recurrence of fractures or deformities, telescopic rod extensions, and Musculoskeletal Tumor Society (MSTS) scores were meticulously recorded during these visits.

### Statistical Analysis

Statistical evaluations were conducted using SPSS 24.0 software. Measured data were expressed as mean ± standard deviation. The paired T-test was employed for intergroup comparisons. A *p*-value < 0.05 was considered indicative of statistical significance.

## Results

### Patient Demographics and Outcomes

The patient cohort comprised 3 males and 1 female, with a median age of 9.5 years (range: 8-11 years). Three lesions were located in the left tibia, while one was in the right tibia. The median lesion segment length was 14.6 cm (range: 9-21.5 cm), with the lesion length ratio averaging 62.3% (range: 50-75%). Among the patients, three (Cases 1, 2, and 3 as per Table 1) had previously undergone curettage and ESIN fixation. One patient (Case 4) had an open biopsy performed and subsequently utilized a brace for protection. Each of the four patients experienced lesion recurrence and rapid lesion progression, with significant anterior bowing averaging an angle of 32 (range: 20 -48) (Fig. 1a, b). The median follow-up duration was 29 months (range: 24-34 months). Radiographic assessments revealed a median time to complete union of 3 months (range: 2.5-4.5 months). Infection developed in one patient, who responded favorably to oral antibiotic administration as well as dressing changes. Another case involved unintentional displacement of the upper section of the telescopic rod without requiring additional medical interventions. According to the modified Clavien-Dindo-Sink surgical complications grading system, the patients were divided into i to ii grades. At the final follow-up, all cases exhibited a limb length discrepancy (LLD) averaging 7.5 mm (range: 5-10 mm). The average extension of the telescopic rod was 10.5 mm (range: 2-20 mm), with one instance of telescopic rod displacement. There were no reported refractures or bowing deformities (Fig. 1e, f). All patients achieved full range of motion in both the knee and ankle joints post-operatively. By the final follow-up, every patient had resumed their routine daily activities without discomfort. The postoperative MSTS score was 26.5 ± 1.12, a significant improvement from the preoperative score of 12.3 ± 1.09 (*p*<0.05).

## Discussion

Osteofibrous dysplasia (OFD) is characterized by osteolysis in the cortex, peripheral sclerosis, dilation and thinning of the cortical surface, and narrowing of the marrow cavity. It is a rare, benign fibroosseous lesion that predominantly occurs in the tibia of children [1]. While often asymptomatic, it can induce pain and deformity [2, 5]. Although OFD bears clinical and radiologic similarities to adamantinoma (ADM), the direct association between the two, especially considering the malignant nature of ADM, remains a subject of debate. Some researchers suggest that OFD could be a precursor to adamantinoma or even develop secondary to adamantinoma degeneration, prompting recommendations for radical surgical measures like wide excision or amputation due to the suspected ADM association [4]. Conversely, others argue that the risk of OFD transforming to ADM appears low in clinical practice [6, 8, 9]. Recent longitudinal studies of both unilateral and bilateral OFD cases extending into adulthood have not identified any instances of malignant transformation [9]. We firmly believe that open biopsy is an essential part of patient management, recommending it for all patients to exclude the presence of adamantinoma and other bone tumors. In this cohort of 4 patients, we conducted pathological examinations during surgery to achieve clear pathological results.

Treatment strategies should be tailored to the clinical and anatomical specifics of the lesion. Observation might be best for children with minor symptoms, such as occasional pain or slight swelling. In contrast, surgical interventions are advised for those presenting with persistent pain, pathological fractures, or worsening tibial deformity [5]. All our studied cases were exceptional OFD examples. All four manifested significant bowing deformity with extensive lesions. The median lesion length was 14.5 cm (9-21.5 cm) with a lesion length ratio of 63.2% (50-75%) and an anterior bowing angle averaging 32° (20°-48°). Clinicians face two primary challenges regarding the natural history of anterior tibial bowing: the self-worsening nature of the bowing deformity and the very low spontaneous healing probability of tibial fractures, which also complicates surgical treatments [10].

Multiple studies posit that the primary objective of OFD treatment is lesion resection to minimize recurrence. This approach has proven effective for small lesions or those limited to a single cortex. Curettage and allograft treatment of OFD have yielded satisfactory outcomes and low recurrence rates as documented by several authors [5, 11]. For extensive lesions causing deformity, most researchers recommend extra-periosteal resection, which, regardless of the reconstruction method employed, can result in significant bone defects and may pose high surgical risks and complications [5, 6].

Lee et al. [4] presented five cases of OFD with expansive lesions, treated with extra-periosteal resection followed by bone transport using the Ilizarov technique. Although all patients achieved symptom and disease remission, the distraction osteogenesis process compelled patients to endure external fixators for several months, a challenge many found hard to accept. Moreover, three patients required further surgery due to nonunion at the docking site, while two experienced pin-site infections.

Another study by Yunan Lu [5] reported five OFD cases (mean age: 8.2 years; range, 2 to 12) of the tibia with extensive lesions. These underwent extra-periosteal excision and primary bone transport using an Ilizarov external fixator. Complications recorded included one case of delayed union, two of pin-site infection, and one of joint stiffness. Given the higher complication rate and elevated costs, Lu advises selective utilization of this technique.

In Dala-Ali's report [6], 19 lesions (extensive, recurrent, or progressive) underwent wide excision, and three distinct reconstruction methods addressed bone defects: bone transport, fibular graft, and megaprosthesis. Bone transport presented with the highest incidence of tibial nonunion (4/6), all of which needed re-grafting. One case required supplementary stabilization using a plate, and another with an external fixator. Fibular graft complications included recurrence (2/10) and nonunion (2/10). Notably, while the free vascularized fibula graft is a globally recognized method for filling bone defects, donor site complications persist.

Dong Li [12] documented 12 patients treated with extraperiosteal segmental excision and reconstruction using liquid nitrogen-treated recycled autograft and allograft to address bone defects. The median resected segment length was 8 cm (range: 5-11 cm). Follow-up radiographs indicated a median complete union time of 9 months (range: 6-15 months). This prolonged healing time necessitated extended external fixation immobilization.(The complications and surgical burden after different methods are presented in Table 3).

**Table 3:** Complications and surgical burden after different methods

Literature	Total number	Treatment	Reconstruction Methods	Complication
Lee [4]	5	Extra-periosteal resection	Bone transport with Ilizarov	Nonunion 3 Pin-site infections 2
Yunan Lu [5]	5	Extra-periosteal excision	Bone transport with an Ilizarov external fixator	Delayed union 1 Pin-site infection 2 Joint stiffness 1
Dala-Ali [6]	19	Wide excision	Bone transport	Tibial nonunion 4/6
			Fibular graft	Recurrence 2/10 Nonunion 2/10
			Megaprosthesis	Loosening requiring Revision 1/3
Dong Li [12]	12	Extraperiosteal segmental excision	Liquid nitrogen-treated recycled autograft with locking plates	Delayed healing 2 Median union time 9 months

As OFD is self-limiting, stabilizing with bone maturity, contemporary research proposes focusing on deformity correction rather than lesion treatment. In a 2022 study, Dala-Ali [6] monitored 101 tibial OFD patients over an average of 5.65 years. The findings certified OFD as benign, with minimal progression and no malignant transformations. Surgery, Dala-Ali suggests, should prioritize angular deformity. In a corroborating study, Daniel Westacott [7] observed 28 tibial OFD cases, noting lesion stability with bone maturity and no malignancy signs. Westacott further promoted minimally invasive techniques to restore tibial force lines and functionality over broad lesion removal.

Similar to the views of these authors, the main purpose of our treatment is to correct angular deformity rather than the lesion.In this paper, we report for the first time the use of minimally invasive tibial osteotomy, and TR fixation to correct anterior bowing deformity and maintain alignment of the tibia. Compared with lesion resection and bone reconstruction, this surgical method has less trauma, faster healing, avoids large bone defects and difficult bone reconstruction , and is more acceptable to patients and their families.

It's crucial to differentiate OFD's anterior bowing deformity from the congenital tibial variant. The latter often precedes congenital tibial pseudarthrosis and frequently associates with type I neurofibromatosis. Given congenital anterior bowing's unique nature, post-osteotomy complications like non-union and pseudarthrosis are common, rendering osteotomy contraindicated for congenital tibial bowing deformities [13, 14]. Few studies discuss potential nonunion or pseudarthrosis following tibial osteotomies in OFD-affected children. In our patient group, osteotomies occurred within the tibial tumor lesion, followed by TR fixation continuity restoration. Subsequent assessments showed successful osteotomy end healing in four patients, averaging a bone healing duration of 3 months (range: 2.5-4.5 months), with no instances of nonunion or pseudarthrosis.

In the case report by Nakahara H [15], a 6-year-old child with OFD exhibiting tibial varus underwent treatment using an intrafocal orthopaedic locking plate for internal fixation. The 3-year follow-up indicated positive outcomes, with no observed deformity, implant failure, or progression of the lesion. The patient demonstrated the ability to run and returned to daily activities without restrictions. However, given the limited follow-up duration, concerns remain regarding potential re-fracture as the child's tibia grows. Contrasting Nakahara H's approach, our study opted for TR fixation. TR has been extensively documented as a secure and durable technique for pediatric deformity correction surgery. It has seen successful applications in the deformity correction of osteogenic imperfections in the femur and tibia and in CROSS-fusion of congenital pseudarthrosis of the tibia in children [16, 17]. However, its use in children with OFD remains unreported. In our study, we detail the application of TR in patients undergoing tibia osteotomy to restore bone alignment. The subsequent outcomes from our cases underscored TR's capability to consistently maintain the tibia's mechanical axis, adapt to the child's growth, and act as a preventive measure against fractures. Yunan Lu [5] mentioned the use of ESIN to preserve the tibial axis post-lesion removal in OFD patients, which provided substantial support. However, with the tibia's growth, ESIN tends to displace from the tibia's ends, necessitating a subsequent procedure for its replacement as the child ages. For the OFD of anterior arch deformity described in this paper, it is preferable to use center-based internal fixation in order to maintain the correct alignment of the tibia and prevent recurrence of the deformity. The telescopic rod chosen plays a crucial role in maintaining proper tibial alignment while allowing for growth and development. Difficulty arises regarding removal or replacement, necessitating proactive follow-up schedules, which ultimately enables early intervention in case of any adverse events. Longer observation times enhance the possibility of detecting potential secondary complications before they become severe.

All four patients in our study displayed LLD with an average extremity length discrepancy of 7.5 mm (ranging from 5-10 mm). This discrepancy may be attributed to the tibia's excessive growth following repeated pathological fractures and partial lesion area resection during surgeries. One patient benefited from insole correction, while the others underwent observation.

The MSTS score, initially formulated for evaluating post-malignant tumor reconstruction functionality [18], was repurposed in our study to assess benign tumors. Its components, including pain, gait, orthosis use, walking capability, function, and patient satisfaction, offer invaluable metrics for clinicians evaluating lower limb diseases. 

We believe the MSTS score is apt for gauging the prognosis of OFD in children. Our surgical procedure significantly enhanced children's quality of life, eliciting heightened satisfaction from parents.

However, our research has its constraints. Firstly, the disease's rarity led to a smaller patient sample size, coupled with the study's retrospective nature. Secondly, the relatively short follow-up duration for the children necessitates extended observation to discern long-term effects until bone maturation.

In conclusion, minimally invasive tibia osteotomy combined with telescopic rod osteosynthesis, without lesion resection, proves effective for treating bowing deformities resulting from osteofibrous dysplasia. This approach circumvents the creation of sizable bone defects and intricate bone reconstructions, markedly enhancing limb functionality and the affected children's quality of life.

## Data Availability

The datasets generated and/or analysed during the current study are not publicly available due to limitations of ethical approval involving the patient data and anonymity but are available from the corresponding author on reasonable request.

## Abbreviations

OFD: Osteofibrous dysplasia
TR: telescopic rod
LLD: limb length discrepancy
